# Robust Stimulation of W1282X-CFTR Channel Activity by a Combination of Allosteric Modulators

**DOI:** 10.1371/journal.pone.0152232

**Published:** 2016-03-23

**Authors:** Wei Wang, Jeong S. Hong, Andras Rab, Eric J. Sorscher, Kevin L. Kirk

**Affiliations:** 1 Gregory Fleming James Cystic Fibrosis Research Center, University of Alabama at Birmingham, Birmingham, AL, 35294, United States of America; 2 Department of Cell, Developmental, and Integrative Biology, University of Alabama at Birmingham, Birmingham, AL, 35294, United States of America; 3 Department of Pediatrics, Emory University, Atlanta, GA, 30322, United States of America; Indiana University School of Medicine, UNITED STATES

## Abstract

W1282X is a common nonsense mutation among cystic fibrosis patients that results in the production of a truncated Cystic Fibrosis Transmembrane Conductance Regulator (CFTR) channel. Here we show that the channel activity of the W1282X-CFTR polypeptide is exceptionally low in excised membrane patches at normally saturating doses of ATP and PKA (single channel open probability (P_O_) < 0.01). However, W1282X-CFTR channels were stimulated by two CFTR modulators, the FDA-approved VX-770 and the dietary compound curcumin. Each of these compounds is an allosteric modulator of CFTR gating that promotes channel activity in the absence of the native ligand, ATP. Although W1282X-CFTR channels were stimulated by VX-770 in the absence of ATP their activities remained dependent on PKA phosphorylation. Thus, activated W1282X-CFTR channels should remain under physiologic control by cyclic nucleotide signaling pathways *in vivo*. VX-770 and curcumin exerted additive effects on W1282X-CFTR channel gating (opening/closing) in excised patches such that the P_o_ of the truncated channel approached unity (> 0.9) when treated with both modulators. VX-770 and curcumin also additively stimulated W1282X-CFTR mediated currents in polarized FRT epithelial monolayers. In this setting, however, the stimulated W1282X-CFTR currents were smaller than those mediated by wild type CFTR (3–5%) due presumably to lower expression levels or cell surface targeting of the truncated protein. Combining allosteric modulators of different mechanistic classes is worth considering as a treatment option for W1282X CF patients perhaps when coupled with maneuvers to increase expression of the truncated protein.

## Introduction

Cystic fibrosis (CF) is caused by loss-of-function mutations in an epithelial anion channel, the cystic fibrosis conductance regulator (CFTR). CF is a multi-organ disease that in severe cases leads to recurring bacterial infections in the airways and progressive lung failure. Over 1000 CFTR mutations have been identified in the CF population with different mutations limiting CFTR function by different mechanisms [1]. W1282X is among the more common CF-causing mutations being quite prevalent in Ashkenazi Jews [2]. The W1282X mutation, which is associated with severe CF, presumably reduces CFTR function by two complementary mechanisms. First, this nonsense mutation decreases the steady state levels of CFTR mRNA and consequently the amount of CFTR protein by a process termed nonsense-mediated mRNA decay (NMD) [3]. Second, those W1282X-CFTR polypeptides that are synthesized probably have low channel activity because they are truncated in the second cytosolic nucleotide binding domain, NBD2 (although to our knowledge this point has not been quantitatively established). CFTR channel opening is strongly dependent on the binding of two ATP molecules at the interface of an NBD1-NBD2 dimer [4, 5] subsequent to phosphorylation of its regulatory domain (R domain) by cyclic nucleotide dependent kinases (e.g., PKA [6]. The truncated W1282X-CFTR polypeptide lacks regions of NBD2 that contribute to forming each of the two ATP binding pockets at this dimer interface (e.g., the Walker B and signature sequence motifs).

One proposed strategy for treating patients with this and other nonsense mutations is to develop drugs that promote the transcriptional read through of stop mutations and the consequent synthesis of the full length CFTR polypeptide. This strategy has shown some initial promise but has not yet developed to the point of being clinically effective [7, 8]. Here we tested the hypothesis that the truncated W1282X-CFTR polypeptide can function as a PKA-regulated anion channel with high activity when treated with allosteric modulators of CFTR channel gating. If true, an alternative therapeutic strategy that combines such modulators with approaches to increase the synthesis of the truncated polypeptide (e.g., NMD inhibitors) also would be worth considering. This hypothesis is based in part on previous reports by us and others that engineered CFTR truncation mutants that lack all of NBD2 are synthesized and trafficked to the surfaces of transfected cells (e.g., HEK-293T cells) where they exhibit low but detectable channel activity [9, 10]. The channel activities of these NBD2 deletion constructs are strongly enhanced by two small molecule activators of CFTR (also called CFTR potentiators); the dietary compound curcumin [9] and the FDA-approved ivacaftor or VX-770 [11, 12]. These compounds appear to function as allosteric modulators that circumvent ATP binding defects by promoting ATP-free channel activity. Finally, there is evidence that the truncated W1282X-CFTR polypeptide can traffic to the surfaces of polarized epithelial monolayers where its activity is enhanced by VX-770; i.e., when heterologously expressed in CFBE41o^-^ airway cells [13]. The latter Ussing chamber results provide indirect estimates of CFTR function rather than quantitative measures of W1282X-CFTR channel activity. But taken together the above findings raise the possibility that pharmacologic strategies to enhance the channel activity of the truncated W1282X-CFTR polypeptide might be feasible.

Here we confirm that the single channel open probability (P_o_) of W1282X-CFTR in excised membrane patches is exceptionally low under standard activation conditions (< 0.01). Remarkably, the P_o_ of this truncation mutant can be increased to near unity by the combined addition of VX-770 and curcumin. These activators affect W1282X-CFTR channel gating by different mechanisms such that their effects are additive in excised membrane patches as well as in polarized FRT epithelial monolayers (Ussing chamber experiments). The W1282X-CFTR-mediated currents across FRT monolayers that were stimulated by these compounds were small relative to wild type CFTR (ca 5%), however, indicating that other factors are limiting in this model epithelium (e.g., protein synthesis or cell surface targeting). Our findings support the feasibility of a pharmacologic approach that combines CFTR potentiators of different mechanistic classes to activate the W1282X-CFTR channel. This approach might be therapeutically beneficial when coupled with strategies to increase production of the truncated W1282X-CFTR protein.

## Materials and Methods

### DNA constructs

CFTR cDNAs were cloned into either pcDNA3 or pcDNA5/FRT (Thermo Fisher) under the control of the CMV promoter. V1198 in CFTR cDNA was replaced with a termination codon in Δ1198-CFTR [9] and the rest of the CFTR cDNA was deleted from the vector. W1282X contains a mutation found in a patient (c.3846G>A): E designates a W1282X cDNA that was truncated following the codon and P designates a W1282X cDNA that contains the X mutation and the rest of the CFTR cDNA.

### Cell Culture and DNA Transfections

HEK-293T cells were cultured in Dulbecco’s modified Eagle’s Medium (DMEM; Invitrogen) supplemented with 10% fetal bovine serum (FBS) and transiently transfected with wild type (WT), W1282X or Δ1198- CFTR cDNA as described previously [9, 14, 15]. Cells transfected with WT or mutant CFTR were grown for 24–72 h at 37°C in DMEM plus 10% FBS without antibiotics prior to patch clamp experiments. Isogenic stable Fisher Rat Thyroid (FRT) cells expressing WT- or W1282X-CFTR were generated using the Flp^™^-in system (Thermo Fisher Scientific) following the manufacturer’s protocol. Briefly, FRT cells with a genomic Flp recombinase target site were established by transfection with pFRT/lacZeo plasmid. The host cell clones were screened for single copy integration of the Flp target site by southern blot analysis and a flp4 clone was used for generating subsequent CFTR-expressing lines. Isogenic cell lines expressing wild-type or W1282X CFTR were generated by Flp recombinase mediated targeted integration of the CFTR expression cassette into the target site. Clonal cell lines were isolated and screened for correct insertion by assaying for loss of β-galactosidase activity. Single copy insertion of CFTR cDNA was tested by RT-PCR based on the absolute quantification method (ABI). FRT cells were cultured in Ham’s F-12, Coon's Modification (Sigma) with 5% FBS and used for unitary current recordings and Ussing chamber experiments.

### Western Blots

FRT cells expressing CFTR were scraped from plates and lysed in RIPA with Halt protease inhibitor cocktail (Thermo Fisher). Protein was quantitated using the BCA assay (Thermo Fisher), samples were mixed with 4× sample buffer, and incubated at 37°C for 10 minutes. Equal amounts of protein (40 μg) were loaded into each lane, resolved by 8% SDS-PAGE, and blotted onto Nitrocellulose membranes. Blocking was with 5% dry milk in PBS plus 0.1% Tween 20 followed by incubation with 10B6.2 mouse anti-CFTR NBD1 primary antibody (1:500; CFTR Folding Consortium) for 2 hours at room temperature, and subsequent goat anti-mouse HRP conjugated secondary antibody (1:5000; Dako) for 1 hour at room temperature. α-tubulin was probed as a loading control (mouse anti-tubuin antibody, Genetex). Labeled proteins were detected using SuperSignal West Femto (CFTR) or Pico (tubulin) ECL kit (Thermo Fisher) and visualized with a Chemidoc (BioRad). CFTR and tubulin bands were quantitated using ImageLab sortware (BioRad). Intensities of CFTR bands were normalized against α-tubulin within the same lanes.

### Patch clamp Recordings and Data Analysis

Macroscopic and unitary currents were recorded in the excised, inside-out configuration using previously described patch clamp techniques [9, 14, 15]. Patch pipettes were pulled from Corning 8161 glass to a tip resistance of 1.5–3.0 MΩ for macroscopic recordings and 3–10 MΩ for unitary current recordings. Pipette and bath solutions were identical and contained in mM: 140 N-methyl-D glucamine, 3 MgCl_2_, 1 EGTA, and 10 TES, adjusted to pH 7.3 with HCl. All patch clamp experiments were performed at room temperature (21–23°C). CFTR channels were activated by 1.5 mM Mg-ATP and 110U/ml PKA catalytic subunit in the cytosolic bath. Macroscopic currents were recorded using a ramp protocol (± 80 mV) with a 10 s time period and analog filtered at 20 Hz. Patches were held at ±60 mV for unitary current recordings which were analog filtered at 110 Hz and then digitally filtered at 50 Hz with Clampfit 9.2 software (Axon Instruments).

The single channel open probabilities (P_o_s) of W1282X-CFTR were estimated in membrane patches excised from transiently transfected HEK-293T cells and from the stably transfected FRT cells. The former expression system provided crude P_o_ estimates for W1282X-CFTR channels under control activation conditions before potentiator addition; crude because of high expression levels with many channels per patch. The latter expression system provided more accurate P_o_ estimates because of fewer channels per patch (≤ 8, the limit for analysis by Clampfit) and was used to estimate the P_o_s of W1282X-CFTR channels before and after potentiator addition. For estimating P_o_s in HEK-293T patches, records containing fewer than 8 simultaneous openings prior to the addition of potentiators were selected for analysis. For such records the product of the number of channels in each patch (N) and the single channel open probability (P_o_) for that patch (NP_o_) was estimated using Clampfit 9.2. This product was then divided by the total number of channels (N) in that patch which was estimated from the steady-state macroscopic current measured after potentiator addition; i.e., as N = I/i where I is the steady-state macroscopic current measured after potentiator addition and i is the unitary current at the holding potential of ±60 mV (0.4 pA). This provides a minimal estimate of N (minimal because the calculation assumes a unity P_o_ for potentiator-activated channels) and consequently a maximal estimate of P_o_ before potentiator addition. Single channel opening rates (r, openings/s-channel) and closed time constants (τ_close_ = 1/r, in seconds) also were estimated for the control condition (i.e., before potentiator addition) from r = R/N, where R is the overall opening rate measured by Clampfit.

For the more conventional single channel analysis of W1282X-CFTR channels in FRT patches, only records that contained fewer than 8 simultaneous openings after potentiator addition were analyzed by Clampfit. Open channel burst durations were analyzed by setting a cutoff time of 100 ms to exclude brief intraburst closings. P_o_s were estimated for each condition by dividing the NP_o_ product measured for that condition by the maximal number of simultaneous openings observed after potentiator addition (N). Single channel opening rates (r, openings/s-channel) and closed time constants (τ_close_ = 1/r, in seconds) were estimated for each condition from r = R/N, where R is the overall opening rate measured by Clampfit. Mean burst durations (MBDs, in seconds) were estimated for multichannel records using the cycle time method described by Mathews et al [16] where T is the length of the channel record (in seconds): MBD = [(NP_o_)T]/ (number of openings)]. This method can be used to roughly estimate MBDs for multichannel patches with the assumption that there exists only one population of openings [16].

The averaged data are presented as the means ±S.E. Statistical comparisons were made by performing unpaired t-tests. To avoid contamination of the recording chamber by VX-770 the chamber was washed extensively after each experiment first by scrubbing it with detergent followed by a water rinse and then washing it again in 50% DMSO.

### Ussing Chamber Experiments

FRT cells stably transfected with WT- or W1282X-CFTR were seeded onto 0.4-mm permeable supports (Costar 3470), cultured for 4–5 days as electrically resistive monolayers, and assayed in Ussing chambers as described (13). A serosal-to-mucosal Cl^-^ gradient (120 to 1.2 mM) was imposed and amiloride (100 μM) was added to the mucosal solution to block Na^+^ currents, followed by forskolin (10 μM), VX-770 (5 μM), curcumin (40 μM) and CFTR inhibitor (CFTR-inh172, 10 μM). Each experimental condition was tested on a minimum of three filters.

### Chemicals

VX-770 (Selleck Chemicals) was dissolved in DMSO and stored as a 10 mM stock at -80°C. Curcumin was dissolved in DMSO and stored as a 60 mM stock at -20°C. Mg-ATP stock solution was adjusted to neutral pH and stored at -20°C. All reagents were from Sigma-Aldrich unless indicated.

## Results

### VX-770 robustly stimulates W1282X-CFTR channel activity in excised macropatches

**Fig 1:**
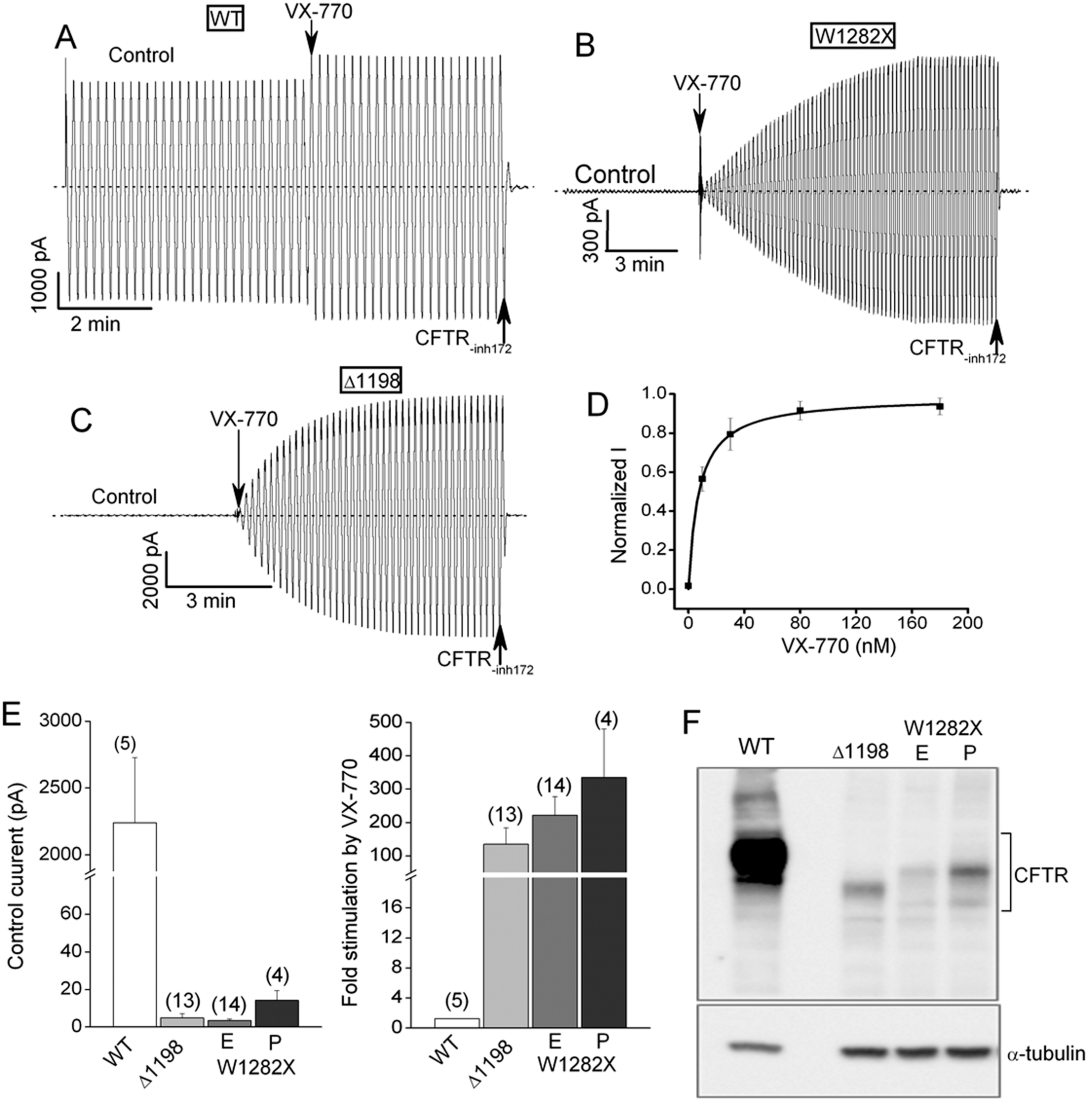
VX-770 robustly activates W1282X-CFTR and Δ1198-CFTR channels in excised HEK-293T macropatches. (A) Control macroscopic current record for an inside-out macropatch excised from an HEK-293T cell expressing WT-CFTR. Ramp protocol (+/- 80 mV); zero current level indicated by the dotted line. Control activation conditions were 110 U/ml PKA and 1.5 mM MgATP in the bath. VX-770 (300 nM) and a CFTR channel inhibitor, CFTR(inh)-172 (10 μM), were added to the bath where indicated. This patch contained several thousand WT-CFTR channels estimated assuming a single channel current of about 0.5 pA at + 80 mV ([9, 14] and Fig 5). VX-770 negligibly stimulated the WT-CFTR current because the channels were nearly maximally active under control condition. (B) Corresponding macroscopic record for an inside-out patch excised from an HEK-293T cell transfected with W1282X-CFTR. Conditions were identical to panel A. Note the very small control current and the marked stimulation by 300 nM VX-770. (C) Corresponding macroscopic record for patch containing Δ1198-CFTR channels. (D) Titration curve for VX-770 activation of W1282X-CFTR channels in excised macropatches. Each symbol represents the mean +/- SE averaged for 6 patches. Conditions were identical to panels A-C. Currents were normalized to the control current before drug addition. Data were fit to a one binding site model with a K_D_ of 10 nM. (E) Mean control currents (left) and fold stimulation by 300 nM VX-770 (right) for the indicated constructs. N’s are indicated in parentheses. Errors are SEs. The E and P versions of W1282X represent an engineered stop mutation and a patient stop mutation, respectively (see Experimental Procedures). (F) Immunoblot showing relative protein levels of the indicated constructs in transfected HEK-293T cells. Total protein loads were identical for each lane (30 μg). The upper band in each lane represents the mature, maximally glycosylated protein.

The macropatch results in Fig 1 show that W1282X-CFTR has very low channel activity in excised membrane patches under control activation conditions but this activity can be strongly stimulated by VX-770. Inside-out macropatches were excised from transiently transfected HEK-293T cells expressing the indicated CFTR constructs. PKA (110 U/ml) and ATP (1.5 mM) were present in the bathing solution at doses that nearly maximally activate wild type CFTR channels (WT-CFTR). Consequently, WT-CFTR exhibited only small current increases following the subsequent addition of 300 nM VX-770 to the bath (Fig 1A). In contrast, W1282X-CFTR (Fig 1B), as well as a previously characterized NBD2 deletion construct (Δ1198-CFTR; Fig 1C), exhibited very small macroscopic currents before VX-770 addition and correspondingly much larger activation responses to this potentiator (mean data in Fig 1E). The stimulation of W1282X-CFTR currents was maximal at doses of VX-770 above 100 nM with an EC_50_ of 10 nM (Fig 1D). The expression levels of the truncated W1282X-CFTR and Δ1198-CFTR polypeptides were less than that for full length WT-CFTR in the transfected HEK-293T cells (see immunoblot in Fig 1F), which presumably contributed in part to their lower absolute control currents. But the much greater relative activation of W1282X-CFTR and Δ1198-CFTR by VX-770 in excised membrane patches indicates that their intrinsic channel activities also must be quite low.

**Fig 2:**
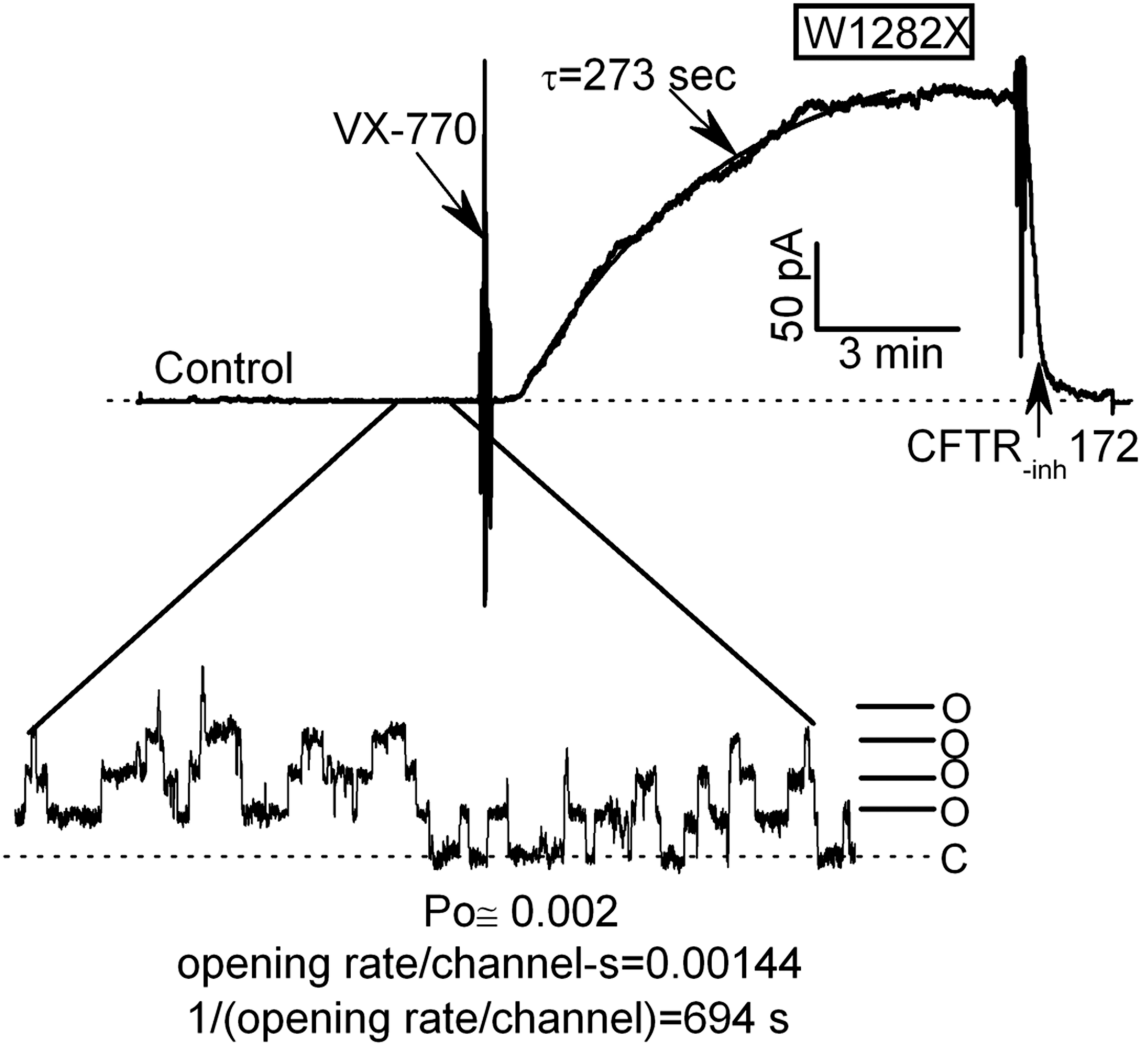
W1282X-CFTR channels exhibit exceptionally low P_o_s in excised HEK-293T patches under control conditions and correspondingly slow activation time courses by 300 nM VX-770. Activation conditions were identical to Fig 1. Holding potential was +60 mV throughout. Inset shows unitary currents under control activation conditions visualized at high gain. The indicated values for the control P_o_, opening rate/channel and closed time constant were calculated for this control record after estimating the total number of channels in the patch from the VX-770 stimulated current. These P_o_ and opening rate values are maximal estimates (see Experimental Procedures). The macroscopic activation time course was fit to a single exponential with a time constant of 273s.

The activation time courses for W1282X-CFTR channels in HEK-293T macropatches treated with 300 nM VX-770 were quite slow in spite of their responses being large in magnitude (Figs 1B and 2). A mean activation time constant of 216 ±29 s (SE; n = 6) was estimated from exponential fits of activation time courses recorded at a single holding potential (see representative ‘gap-free’ record with curve fit in Fig 2). This remarkably slow activation by VX-770 might indicate that W1282X-CFTR channels must open before they bind (or bind tightly) to this compound (see also [17]). In support of this argument, the single channel open probabilities (P_o_s) of W1282X-CFTR in excised HEK-293T patches prior to VX-770 addition were estimated to be less than 0.01 (ranging from 0.001 to 0.005; see Fig 2 and Experimental Procedures). These control P_o_ values were low primarily because of exceptionally slow single channel opening rates ((ranging from 0.0008 to 0.003 openings/channel-s) and correspondingly long closed time constants (ranging from 333 to 1250s, calculated as the reciprocal of the single channel opening rate). Given that W1282X-CFTR channels open so infrequently under control conditions, their slow rate of activation by VX-770 is consistent with the idea that open channels have a higher affinity for this compound (as would be predicted for an allosteric modulator; see Discussion).

### W1282X-CFTR channel activity that is stimulated by VX-770 depends on PKA phosphorylation but not ATP binding

**Fig 3:**
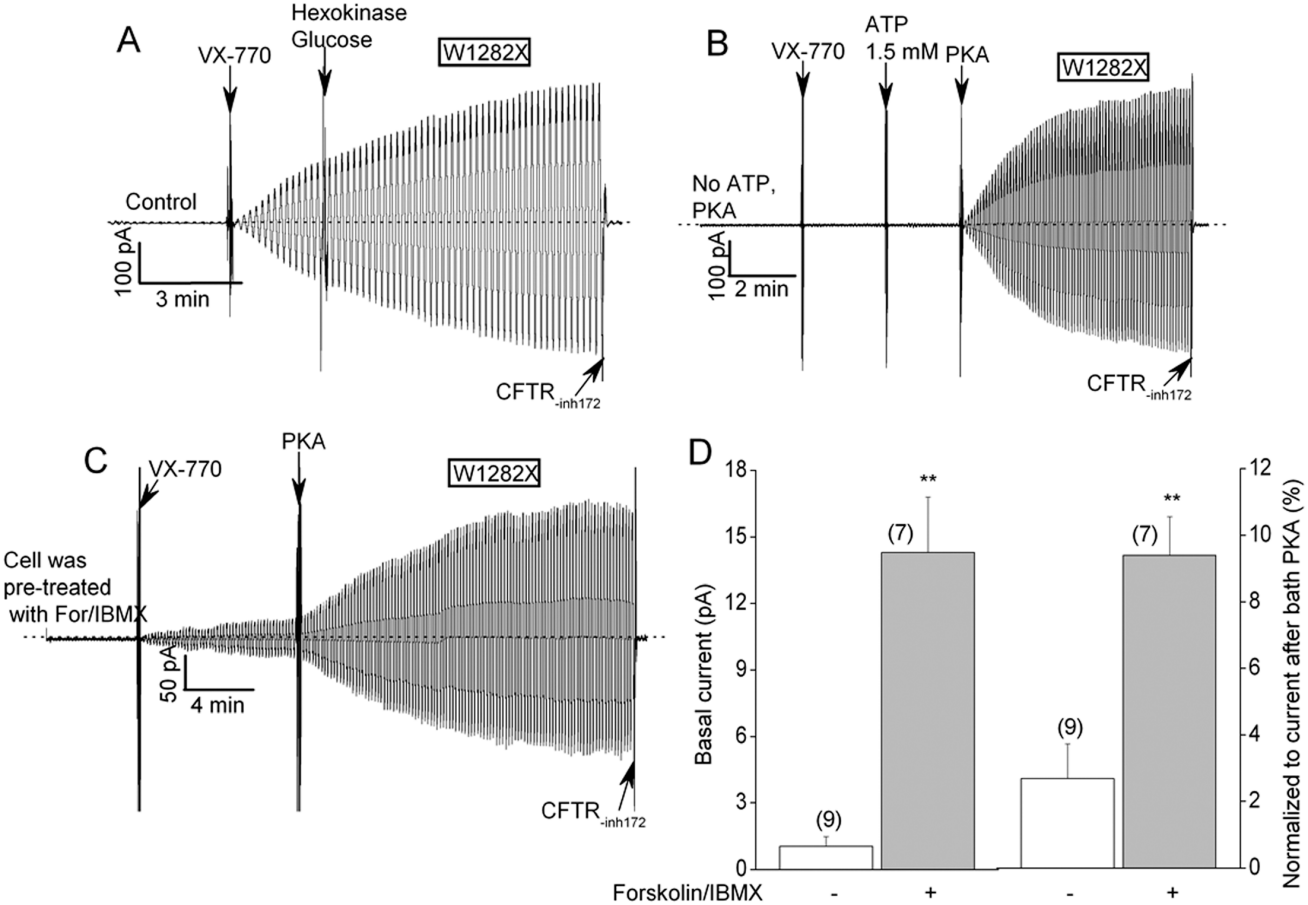
W1282X-CFTR channel activation by VX-770 in HEK-293T macropatches requires PKA phosphorylation but not ATP binding. (A) Macroscopic current record showing that addition of an ATP scavenger to the bath (24U/ml hexokinase/10 mM glucose ([14]) did not inhibit activation of W1282X-CFTR channels by VX-770. Control conditions were identical to Fig 1. (B) Macroscopic current record showing that PKA (110 U/ml) was required for VX-770 activation of W1282X-CFTR. (C) Macroscopic current record showing detectable W1282X-CFTR current in the absence of bath PKA for a patch excised from an HEK-293T cell pre-treated with forskolin (40 μM) and IBMX (100 μM) for 5 mins before excision. 1.5 mM MgATP was present in the bath throughout this experiment. PKA (110U/ml) was added to the bath where indicated. (D) Mean basal currents measured at + 80 mV before bath PKA addition (left) and basal currents normalized to the currents measured after bath PKA addition (right) for macropatches excised from cells pre-treated (or not) with forskolin and IBMX. Errors are SEs. N’s are indicated in parentheses. ** p<0.01 compared to untreated control by unpaired t-test.

Fig 3A shows that the VX-770-stimulated currents that are mediated by W1282X-CFTR in HEK-293T macropatches were insensitive to the addition of an ATP scavenger (hexokinase plus glucose) to the bath. At these doses the ATP scavenger nearly completely inhibits (> 99%) the ATP-dependent currents mediated by WT-CFTR channels [14]. The lack of effect of the ATP scavenger on the W1282X-CFTR currents supports the idea that VX-770 is an allosteric modulator that promotes channel activity in lieu of ATP binding [17, 18]. Conversely, the W1282X-CFTR currents that were stimulated by VX-770 remained strongly dependent on PKA addition to the bath (Fig 3B). The PKA-dependence of W1282X-CFTR activity also was evident in experiments in which HEK-293T cells were pre-treated with forskolin and IBMX prior to patch excision. Patches that were excised from such pre-treated cells exhibited greater current responses to VX-770 when the potentiator was added in the absence of bath PKA as compared to patches that were excised from untreated cells (Fig 3C and 3D). The PKA-dependence of these currents indicates that W1282X-CFTR channels that have been activated by VX-770 remain subject to physiologic regulation; e.g., by cyclic nucleotide signaling pathways.

### Curcumin and VX-770 additively increase W1282X-CFTR channel activity in excised patches

**Fig 4:**
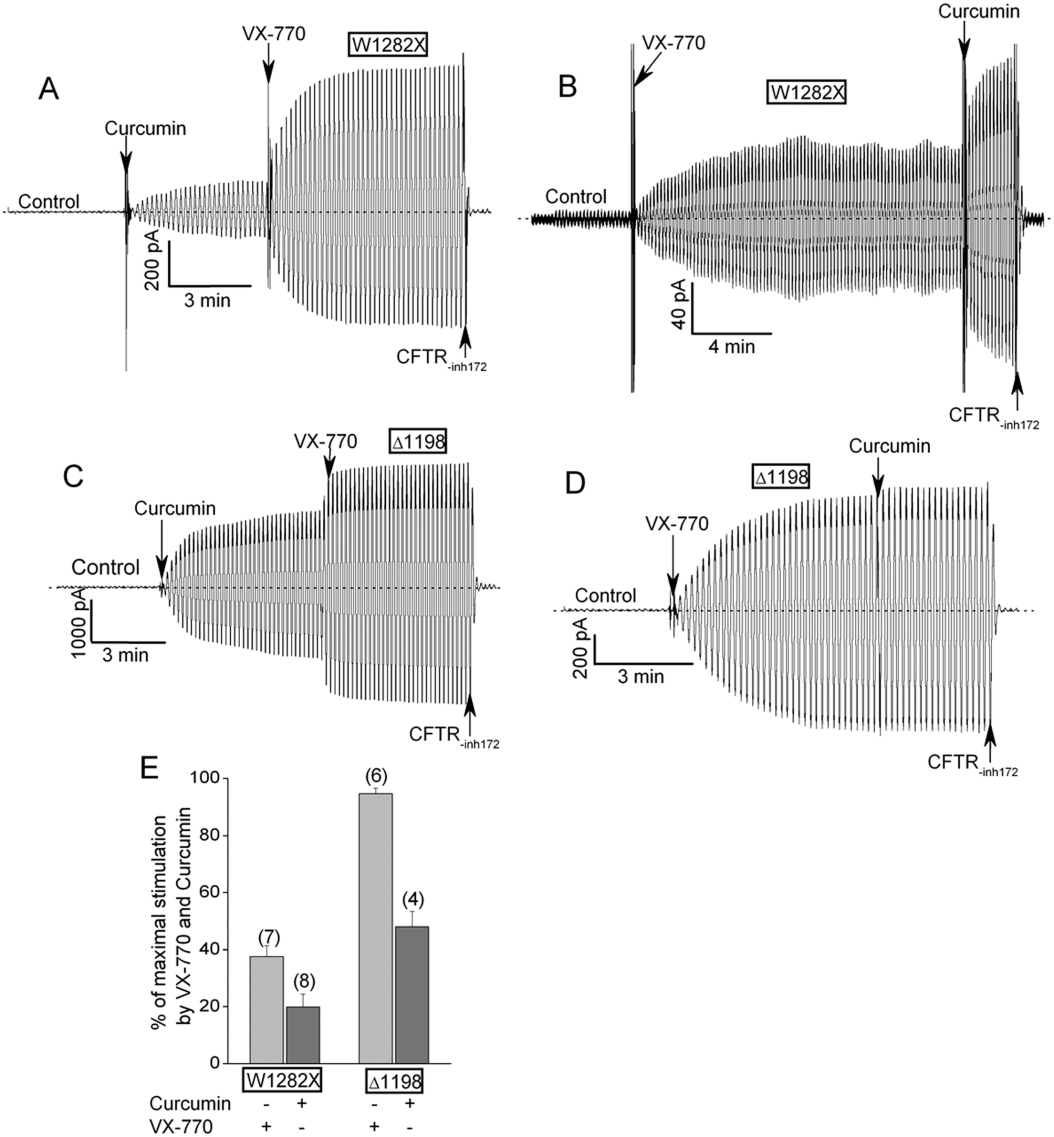
Curcumin and VX-770 additively stimulate W1282X-CFTR currents in excised HEK-293T macropatches. (A) Macroscopic current record showing substantial stimulation of W1282X-CFTR channels by 30 μM curcumin added to the bath prior to VX-770. This curcumin concentration was chosen because it is the maximally effective dose for stimulating Δ1198-CFTR activity [9]. (B) Curcumin strongly stimulated W1282X-CFTR channels after they were first activated by a saturating dose of VX-770 (300 nM). (C, D) Analogous order of addition experiments for Δ1198-CFTR macropatches. (E) Mean percent stimulation of W1282X-CFTR or Δ1198-CFTR current by each compound alone normalized to the total current measured after the addition of both compounds. Errors are SEs. N’s are indicated in parentheses.

Fig 4 shows that W1282X-CFTR channel activity in excised HEK-293T macropatches was also stimulated by 30 μM curcumin, a dietary compound that we showed previously to robustly stimulate Δ1198-CFTR channel activity [9]. Interestingly, 30 μM curcumin and 300 nM VX-770 had additive effects on the macroscopic currents mediated W1282X-CFTR (Fig 4A and 4B). Conversely, 300 nM VX-770 alone nearly maximally activated the currents mediated by Δ1198-CFTR (Fig 4D and 4E). The record in Fig 4B is a clear example of the additive effects of the two compounds on W1282X-CFTR activity (see also mean data in panel E). Curcumin strongly increased the current mediated by this construct well above the level stimulated by a maximally effective dose of VX-770 (300 nM; see VX-770 titration in Fig 1).

**Fig 5:**
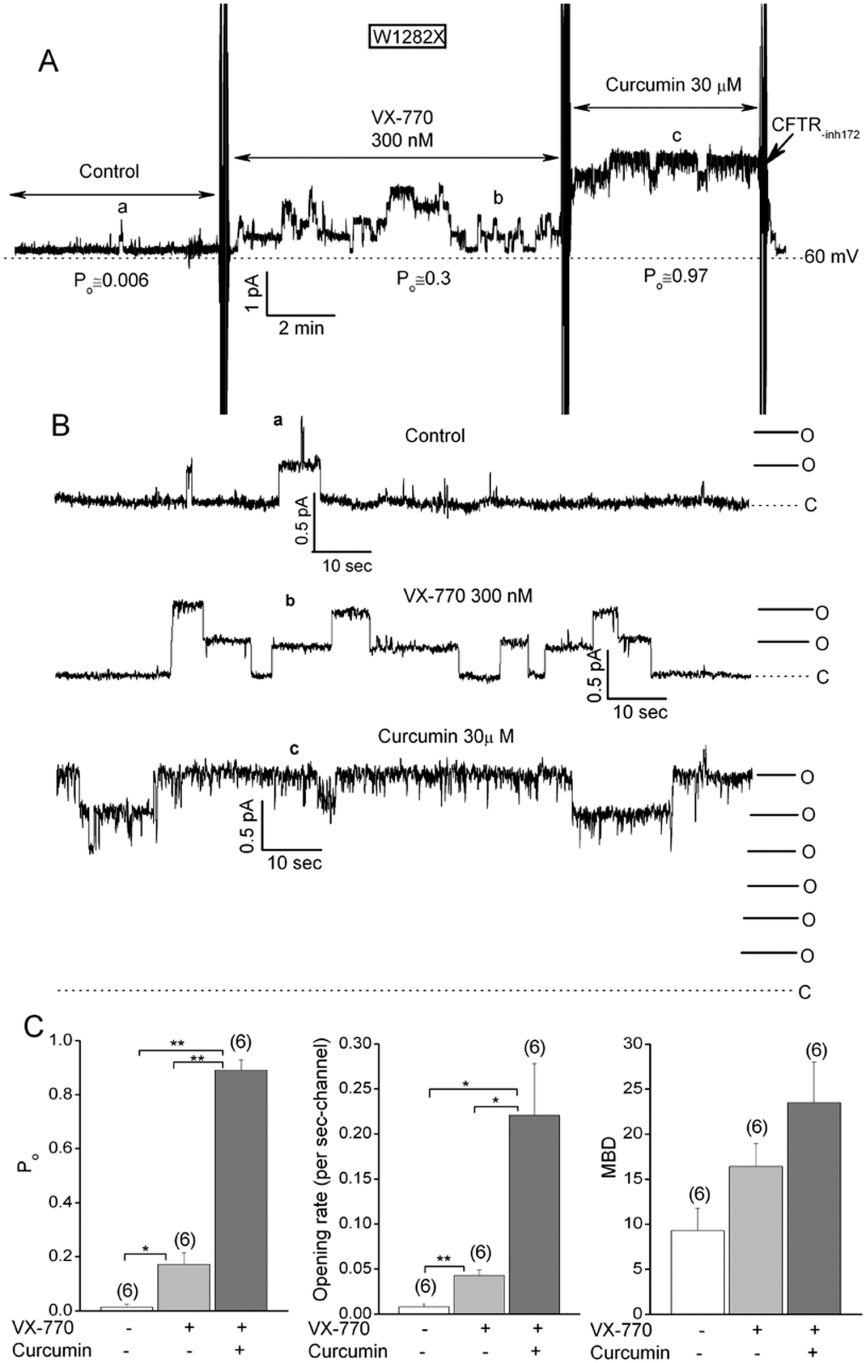
W1282X-CFTR channels in FRT patches are virtually ‘locked open’ by adding curcumin after a saturating dose of VX-770. (A) Multichannel record for inside-out patch excised from FRT cell stably expressing W1282X-CFTR. Control conditions were identical to Fig 1. 300 nM VX-770 and 30 μM curcumin were added where indicated. Holding potential = 60 mV. This patch contained 6 channels. The P_o_ estimated for each condition in this experiment is indicated. (B) Higher gain records for the experiment in panel A. (C) Mean P_o_, single channel opening rate and mean burst duration for W1282X-CFTR channels estimated for the indicated conditions. Error bars are SEs. N’s are indicated in parentheses. *p<0.05 and **<0.01 by paired t-test as indicated.

**Fig 6:**
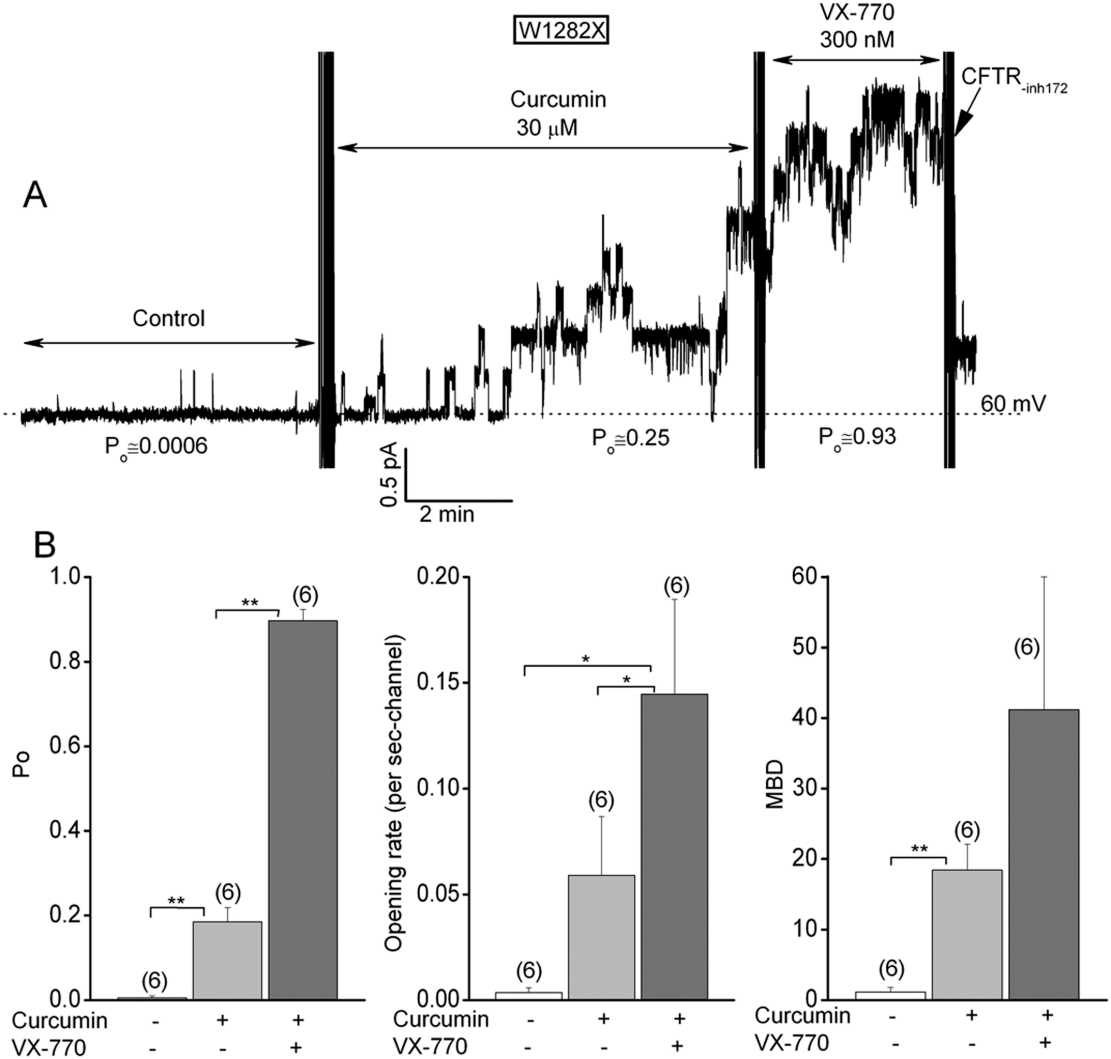
Sequential effects of 30 μM curcumin followed by 300 nM VX-770 on the P_o_s of W1282X-CFTR channels in FRT patches. See Fig 6 for conditions and details. *p<0.05 and **<0.01 (see above).

Single channel experiments were next performed to explore how VX-770 and curcumin exert such additive effects on W1282X-CFTR channel activity (Figs 5 and 6). FRT cells that are stably transfected with W1282X-CFTR were used for these experiments because they express low amounts of W1282X-CFTR protein, which optimizes obtaining patches with sufficiently few channels for single channel analysis after drug addition (< 8 channels). The results in Fig 5 support the following conclusions. First, the P_o_ of W1282X-CFTR channels in excised FRT patches under control activation conditions was exceptionally low (< 0.01); similar to our initial P_o_ estimates for patches excised from HEK-293T cells (Fig 2). Second, the addition of a saturating dose of VX-770 alone (300 nM) increased the single channel P_o_ of W1282X-CFTR to about 0.2; an effect that was due in large part to an increase in the frequency of channel openings (i.e., an increased channel opening rate). Third, the subsequent addition of 30 μM curcumin further increased the P_o_ to near unity both by increasing the opening rate further and by stabilizing those openings as indicated by the increase in mean burst duration.

In reversed order of addition experiments (Fig 6) we observed that 30 μM curcumin alone increased the P_o_ of W1282X-CFTR to about 0.2 by increasing both opening rate and mean burst duration. The subsequent addition of 300 nM VX-770 resulted in W1282X-CFTR channels with exceptionally high P_o_s, which is consistent with the results in Fig 5. The additive effects of the two compounds on the P_o_ of the truncated W1282X-CFTR channel indicates that they probably bind to different sites and affect channel gating by different mechanisms.

### The combination of VX-770 and curcumin stimulates W1282X-CFTR channel activity in polarized FRT epithelial monolayers

**Fig 7:**
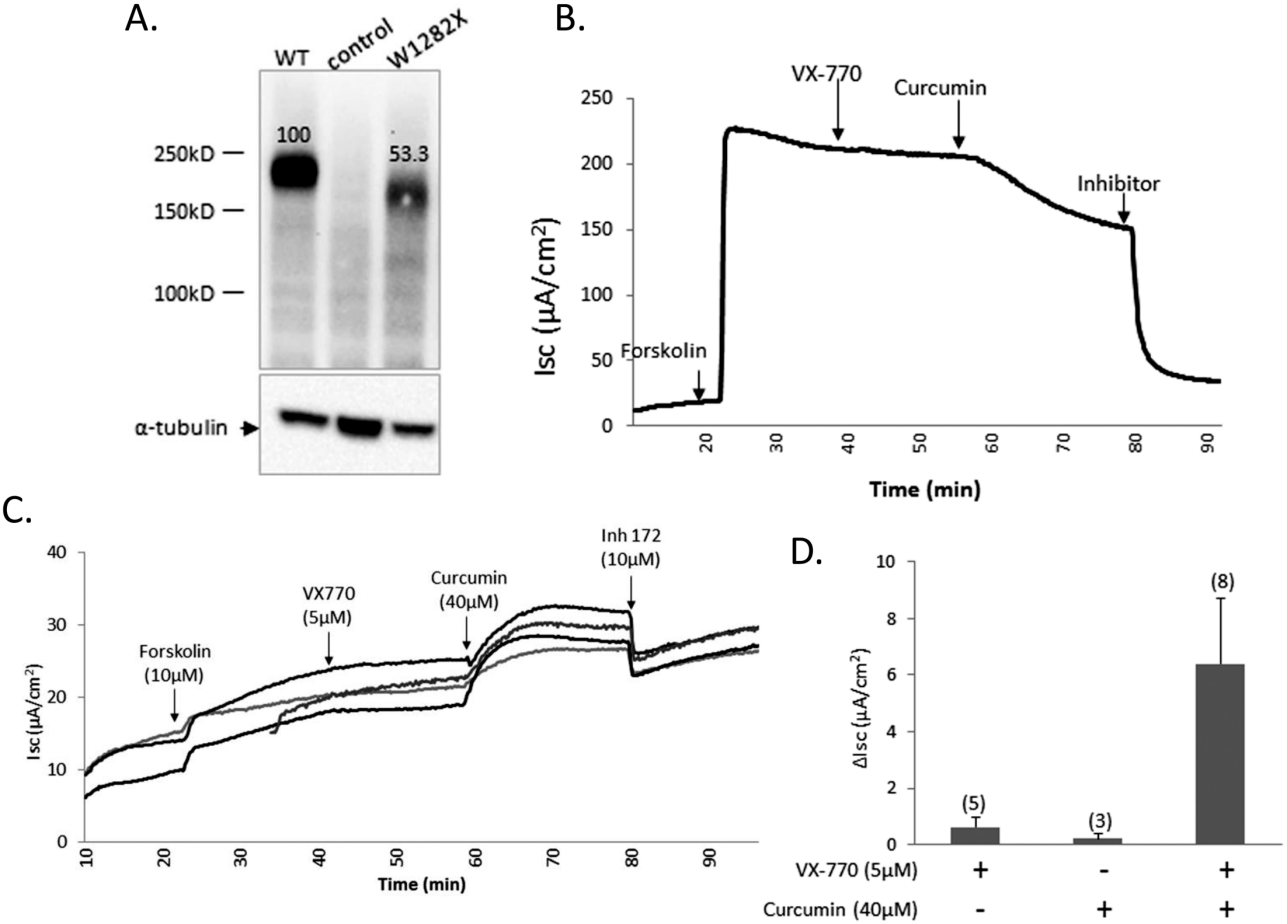
Activation of W1282X-CFTR mediated currents in FRT cell monolayers by the combination of VX-770 and curcumin. (A) Western blot analysis of stably transfected FRT cells. Lane 1: WT-CFTR; lane 2: control FRT cell lysate; lane 3: W1282X- CFTR. The number indicates relative amount of CFTR protein. 40 μg total protein was loaded in each lane. Tubulin controls are shown in the bottom lanes. (B) Short circuit current measurement for FRT cells expressing WT-CFTR (see Experimental Procedures for details). Drug concentrations were 10 μM forskolin, 5 μM VX-770, 40 μM curcumin and 10 μM CFTR(inh)-172, (C) Short circuit measurement for W1282X-CFTR expressing FRT cells. Each trace represents an individual experiment. (D) The bars represent mean (+/- SE) increase in short circuit current above forskolin-stimulated current upon addition of VX-770 and/or curcumin followed by the combination of the two compounds in W1282X-CFTR expressing cells.

To determine if VX-770 and/or curcumin activate W1282X-CFTR channels in intact epithelial monolayers we tested their effects in Ussing chamber experiments performed using stably transfected FRT epithelial cells (Fig 7). FRT cells expressing WT-CFTR served as controls. Monolayers were first exposed to a transepithelial chloride gradient to generate a concentration driving force and then treated with a high dose of forskolin (10 μM) to stimulate the production of intracellular cyclic AMP. FRT monolayers expressing WT-CFTR exhibited very large current increases in response to forskolin (ca 200 μA/cm^2^); not surprisingly, these forskolin-induced currents were much greater than for monolayers expressing W1282X-CFTR (see Fig 7C). The W1282X-CFTR mediated currents were negligibly stimulated by the subsequent addition of 5 μM VX-770 alone (see individual traces in Fig 7C and mean data in Fig 7D). The same was true for the addition of 40 μM curcumin alone (mean data in Fig 7D). These drug concentrations were higher than those used in the excised inside-out patch experiments in case the cytosolic concentrations of these compounds are limited by their cell permeabilities or metabolism. Interestingly, combining the two modulators resulted in a reproducible increase in the W1282X-CFTR mediated currents across FRT monolayers. The stimulatory effect of combining both modulators was easily detectable although the maximal W1282X-CFTR mediated currents were considerably smaller in magnitude when compared to the maximal forskolin-activated currents across WT-CFTR monolayers (3–5%). One factor that likely contributed to the lower currents across the W1282X-CFTR monolayers was a reduced amount of the truncated CFTR protein relative to the wild type protein in the stably transfected FRT cells (ranging from 25–50%; see example immunoblot in Fig 7A). It is also possible that other factors limit W1282X-CFTR activity or its sensitivity to modulators in this model epithelium (e.g., cell surface targeting of the truncated protein or cytosolic access of the compounds). In sum, the Ussing chamber results support the idea that W1282X-CFTR channels are more strongly activated by a combination of allosteric modulators. Such an approach may be therapeutically beneficial when coupled with maneuvers to increase expression of the truncated polypeptide.

## Discussion

The present findings confirm that W1282X-CFTR channels have exceptionally low activities (P_o_ < 0.01) in excised membrane patches at PKA and ATP concentrations that maximally activate WT-CFTR. However, the activity of this truncation mutant can be stimulated robustly and additively by two compounds, VX-770 and curcumin. The low intrinsic activity of W1282X-CFTR is consistent with the normally strong dependence of CFTR opening on the binding of two ATP molecules at the NBD1-NBD2 dimer interface [4, 5]. The truncated W1282X-CFTR polypeptide lacks regions that contribute to forming each ATP binding pocket; namely, the Walker B sequence at ATP binding site 2 (residues 1366 to 1372) and the NBD2 signature sequence that lines site 1 (residues 1346 to 1350). Thus, it is not surprising that this channel has low activity under normal activation conditions although to our knowledge the extent of its gating defect had not been quantitatively established until now. The fact that the single channel activity of W1282X-CFTR was enhanced so markedly by VX-770 and curcumin (P_o_ ≥ 0.9) indicates that pharmacological approaches that improve the activity of the truncated polypeptide may be therapeutically beneficial in some settings.

### VX-770 is an allosteric activator of W1282X-CFTR gating that bypasses the dependence on ATP binding but not PKA phosphorylation

Several observations support the idea that VX-770 is an allosteric modulator of CFTR gating that by definition increases channel activity independently of ATP binding [19–21]. First, we observed that addition of an ATP scavenger to the bath had no effect on the activation of W1282X-CFTR by VX-770 in excised patches. Second, Yeh et al [12] showed that the activity of an engineered NBD2 truncation mutant was substantially elevated by VX-770, as we also observed here for the Δ1198-CFTR construct. Third, Jih et al [17] and Eckford et al [18] earlier reported that this compound increased WT-CFTR activity and the activity of a common CF regulation mutant (G551D) in the absence of ATP. Clearly, this compound bypasses the normal dependence of CFTR gating on ATP binding and NBD dimerization, perhaps by binding to the transmembrane domains [17] where it biases the closed-open equilibrium toward the open state.

The very slow activation of W1282X-CFTR by VX-770 is consistent with such an allosteric mechanism. For any allosteric activation scheme the agonist binds with higher affinity to the active state; i.e., the open conformation in the case of an ion channel [19–21]. The relative binding affinities are determined by the ratio of the open-closed equilibrium constants in the presence and absence of bound ligand [19–21]. We observed that a saturating dose of VX-770 increased the P_o_ of W1282X-CFTR in excised FRT patches by more than 30-fold from less than 0.01 to about 0.2 (Fig 5). This corresponds to a greater than 30-fold difference in the open-closed equilibrium constants (K_eq_s) in the presence and absence of drug (where K_eq_ = P_o_/(1-P_o_)), which predicts a more than 30-fold higher VX-770 binding affinity for the open state assuming a simple two state, one binding site scheme [19–21]. Given these considerations the rate of activation by VX-770 should be limited by the very slow rate of W1282X-CFTR channel opening that was observed under control conditions (i.e., before drug addition; Figs 2 and 5).

VX-770 did not bypass the normal requirement for PKA phosphorylation. W1282X-CFTR channels were not activated by this compound unless they were phosphorylated by PKA in excised patches or in the cells prior to patch excision by pretreatment with forskolin and IBMX. This phosphorylation requirement is consistent with our earlier evidence that the activities of Δ1198-CFTR constructs with gain of function mutations remain PKA-dependent in spite of the fact that they cannot bind ATP or dimerize their NBDs (because they lack NBD2 [14]). Eckford et al [18] also reported that VX-770 only stimulates WT-CFTR channels that have been phosphorylated by PKA. The mechanism by which PKA phosphorylation of the R domain promotes channel opening is unclear and possibly complex. Irrespective of the underlying mechanism the present W1282X-CFTR results and the earlier findings noted above indicate that PKA phosphorylation is an essential regulator of channel opening in lieu of ATP binding or NBD dimerization. This is an important physiologic point when considering the use of allosteric drugs to treat CF patients. CF mutant channels are unlikely to be constitutively active *in vivo* when stimulated by this and presumably other allosteric modulators. Instead, the activities of pharmacologically rescued channels should remain under some level of control by cyclic nucleotide signaling pathways.

### Curcumin and VX-770 additively increase W1282X-CFTR channel activity

W1282X-CFTR channels in excised patches were very strongly stimulated by the combination of VX-770 and curcumin (P_o_ ≥ 0.9). The latter dietary compound was previously shown to increase the activities of Δ1198-CFTR and G551D-CFTR channels in excised macropatches [9]. Sohma et al [22] also reported that curcumin and genistein together exert additive stimulatory effects on G551D-CFTR channel activity. In our excised macropatch experiments we observed that W1282X-CFTR-mediated currents were further and substantially stimulated by curcumin when this compound was added after a saturating dose of VX-770. This additivity was also evident in single channel experiments in which we observed that VX-770 increased the P_o_ of W1282X-CFTR channels to about 0.2 in large part by increasing the rates of channel opening whereas the subsequent addition of curcumin further stabilized those openings and nearly locked open the truncated channel (P_O_ ≥ 0.9). Curcumin and VX-770 each can be classified as an allosteric modulator that stimulates channel activity independently of ATP binding or NBD dimerization. But the additivity of their allosteric effects on CFTR gating indicates that they stimulate channel activity by different mechanisms. The existence of allosteric modulators of different mechanistic classes raises the prospect of combination therapies that exploit such additivity. Curcumin provides one example of a modulator that might be beneficial when combined with VX-770 although it remains to be seen if effective doses of this dietary compound can be achieved *in vivo*. If not, developing more potent analogs or derivatives that activate CFTR channels by the same mechanism as curcumin would be worth considering.

### Therapeutic implication

The main therapeutic implication of the current study is that pharmacologic strategies that promote the activity and the expression of the truncated W1282X-CFTR polypeptide merit consideration (i.e., in parallel with ongoing attempts to promote translational read through of stop mutations [7]). The truncated W1282X-CFTR polypeptide can reach remarkably high single channel activities following treatment with a combination of allosteric modulators. We recognize that a pharmacologic approach that solely increases W1282X-CFTR channel activity may be insufficient if the amount of the truncated polypeptide is severely limiting *in vivo*. The expression of the truncated W1282X-CFTR protein in CF patients is expected to be reduced by NMD, which degrades transcripts that harbor non-native stop mutations [3]. It is also possible that the maturation, stability or plasma membrane targeting of the truncated W1282X-CFTR polypeptide is lower than for the wild type protein. In this regard our Ussing chamber experiments revealed that the combination of VX-770 and curcumin increased W1282X-CFTR-mediated currents in polarized epithelial (FRT) monolayers, but this effect was considerably smaller than for FRT monolayers expressing WT-CFTR (3–5% of the maximal WT-CFTR response). The steady-state expression level of the W1282X-CFTR protein was lower in the FRT cells (25–50% of WT-CFTR), which could contribute in part to the lower functional response in the Ussing chamber experiments. But other factors such as those noted above may limit W1282X-CFTR activity in epithelial tissues as well. If so, allosteric modulators that directly affect channel gating would have to be coupled with maneuvers to increase the steady-state expression of the truncated W1282X-CFTR polypeptide (e.g., NMD inhibitors). The encouraging news is that the latter approaches may not have to be highly effective given how robustly the single channel activity of the truncated W1282X-CFTR polypeptide can be stimulated by a combination of modulators.
